# Supplementary material for: Exploring Plastome Diversity and Molecular Evolution Within Genus Tortula (Family Pottiaceae, Bryophyta)
Source: Plants (Basel). 2025 Sep 8;14(17):2808. doi: 10.3390/plants14172808 (PMC12430682; doi:10.3390/plants14172808)
Supplement: Supplementary file 1 [file plants-14-02808-s001.zip › Figures S1.pdf]

Figures S1. Annotated Chloroplast Genome Maps of Nine *Tortula* Species and *Syntrichia princeps*. The figures are described and mapped in detail as follows:

1. Section *Tortula*

a) *Tortula atrovirens*

High-throughput sequencing of *T. atrovirens*, followed by quality filtering, resulted in the successful assembly of the chloroplast genome with a coverage of 498×. The complete chloroplast genome of *T. atrovirens* is 122,614 base pairs (bp) in length and exhibits a typical quadripartite structure. It consists of LSC region of 84,077 bp, SSC region of 18,639 bp, and a pair of inverted repeats (IR) regions, each 9,949 bp in length. The overall GC content is 28.50 %, with a nucleotide composition of A (35.7 %), T (35.8 %), C (14.3 %), and G (14.2 %)., The chloroplast genome encodes a total of 126 genes, including 81 protein-coding genes, 37 transfer RNAs (tRNAs), and 8 ribosomal RNAs (rRNAs) were identified, consistent with the typical structure of bryophytes plastomes (Figure S1.1).

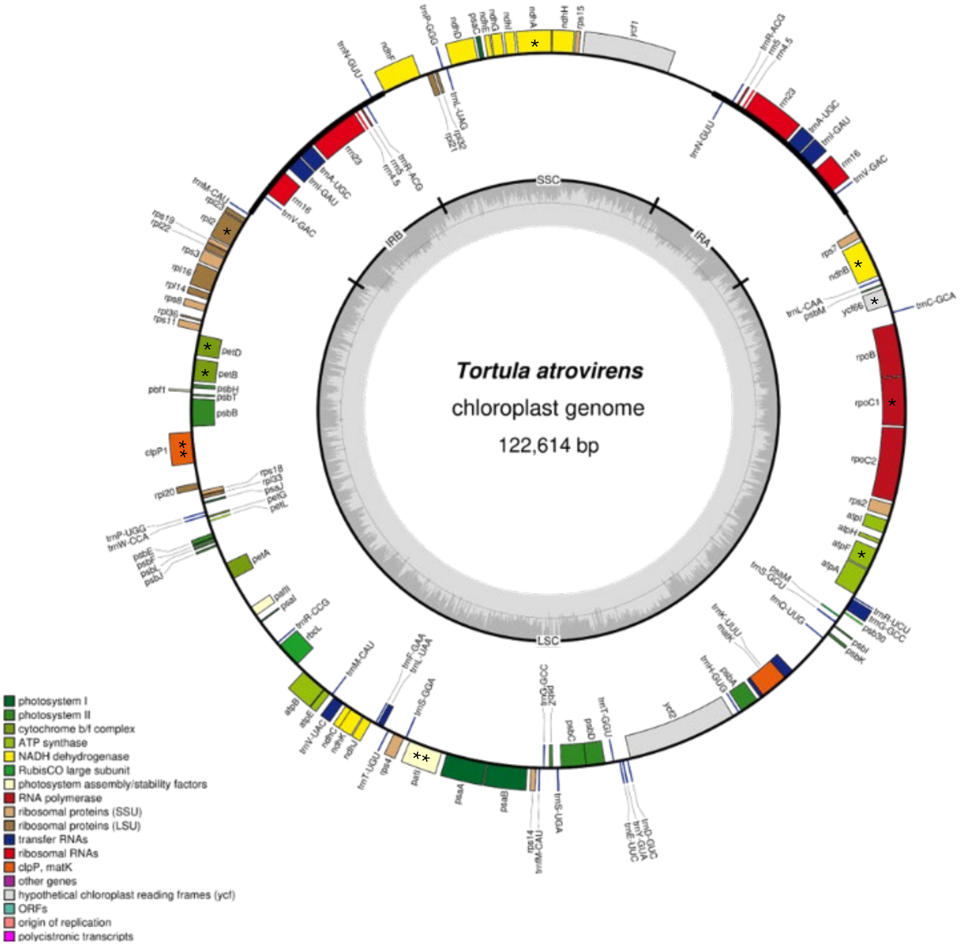

Figure S1.1. Chloroplast genome map of *Tortula atrovirens* with a total length of 122,614 bp. The inner circle represents the guanine and cytosine (GC) content across different regions of the chloroplast genome and is defined by the major chloroplast regions: large single-copy (LSC), small single-copy (SSC), and inverted repeats (IRA and IRB). The outer circle represents the complete chloroplast sequence, with the genes annotated outside the circle to represent forward strand genes and those annotated inside the circle to represent reversed strand genes. All genes are colored according to their functional groups, as indicated in the right part of the figure (* and ** represent genes with one and two introns, respectively).

b) *Tortula brevissima*

High-throughput sequencing of *T. brevissima*, followed by quality filtering, enabled the successful assembly of the chloroplast genome with a coverage of 463×. The complete chloroplast genome of *T. brevissima* is 122,697 (bp) in length and exhibits a typical quadripartite structure. It consists of LSC region of 84,173 bp, SSC region of 18,684 bp, and a pair of IR regions, each 9,920 bp in length. The overall GC content is 28.30 %, with a nucleotide composition of A (35.9 %), T (35.8 %), C (14.2 %), and G (14.1 %). The chloroplast genome encodes a total of 126 genes, including 81 protein-coding genes, 37 transfer RNAs (tRNAs), and 8 ribosomal RNAs (rRNAs) were identified, consistent with the typical structure of bryophytes plastomes (Figure S1.2).

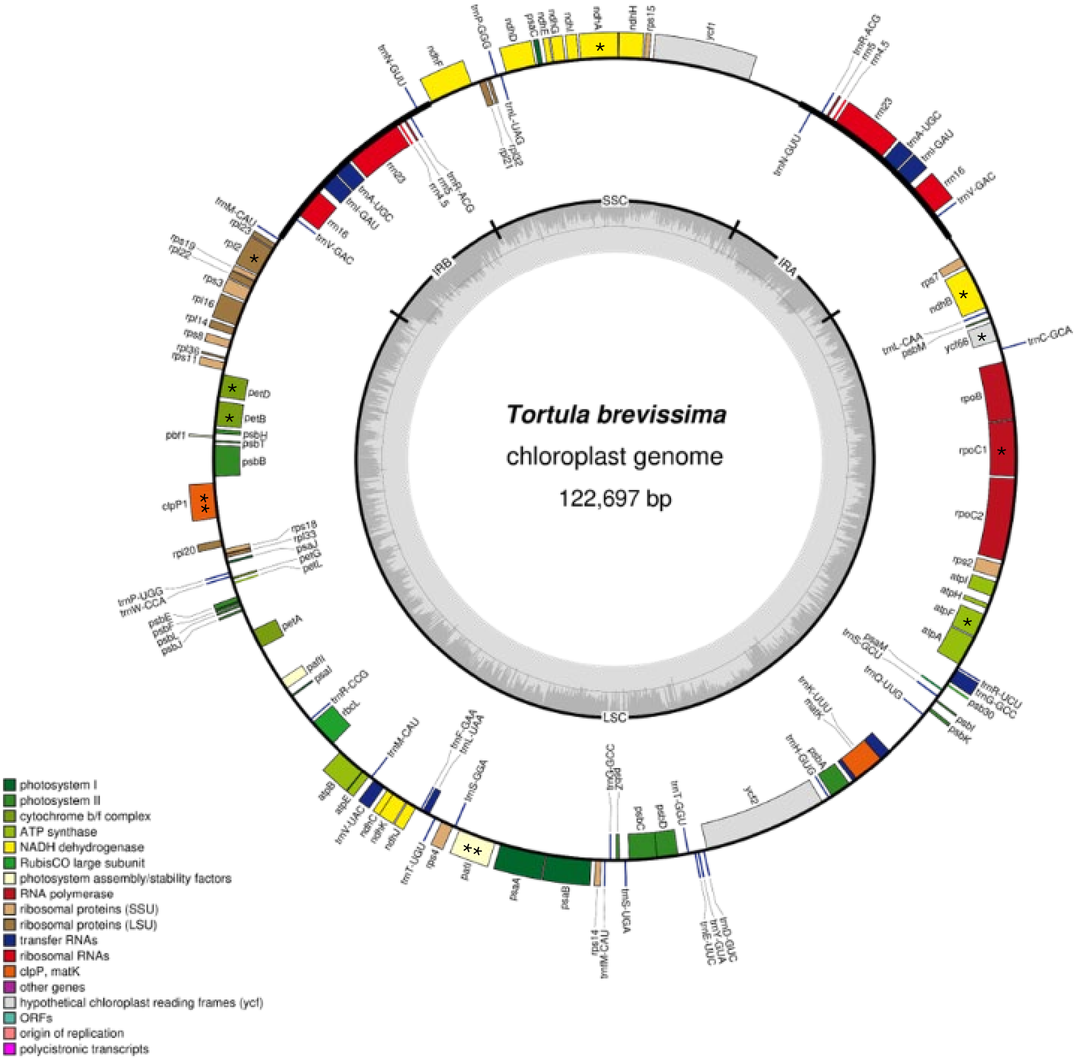

Figure S1.2. Chloroplast genome map of *Tortula brevissima* with a total length of 122,697 bp. The inner circle represents the guanine and cytosine (GC) content across different regions of the chloroplast genome and is defined by the major chloroplast regions: large single-copy (LSC), small single-copy (SSC), and inverted repeats (IRA and IRB). The outer circle represents the complete chloroplast sequence, with the genes annotated outside the circle to represent forward strand genes and those annotated inside the circle to represent reversed strand genes. All genes are colored according to their functional groups, as indicated in the right part of the figure (* and ** represent genes with one and two introns, respectively).

c) *Tortula mucronifolia*

High-throughput sequencing of *T. mucronifolia*, followed by quality filtering, enabled the successful assembly of the chloroplast genome with a coverage of 156×. The complete chloroplast genome of *T. mucronifolia* is 122,530 bp in length and exhibits a typical quadripartite structure. It consists of LSC region of 83,961 bp, SSC region of 18,609 bp, and a pair of IR regions, each 9,923 bp in length. The overall GC content is 28.20 %, with a nucleotide composition of A (35.8 %), T (36 %), C (14.2 %), and G (14 %). The chloroplast genome encodes a total of 126 genes, including 81 protein-coding genes, 37 transfer RNAs (tRNAs), and 8 ribosomal RNAs (rRNAs) were identified, consistent with the typical structure of bryophytes plastomes (Figure S1.3).

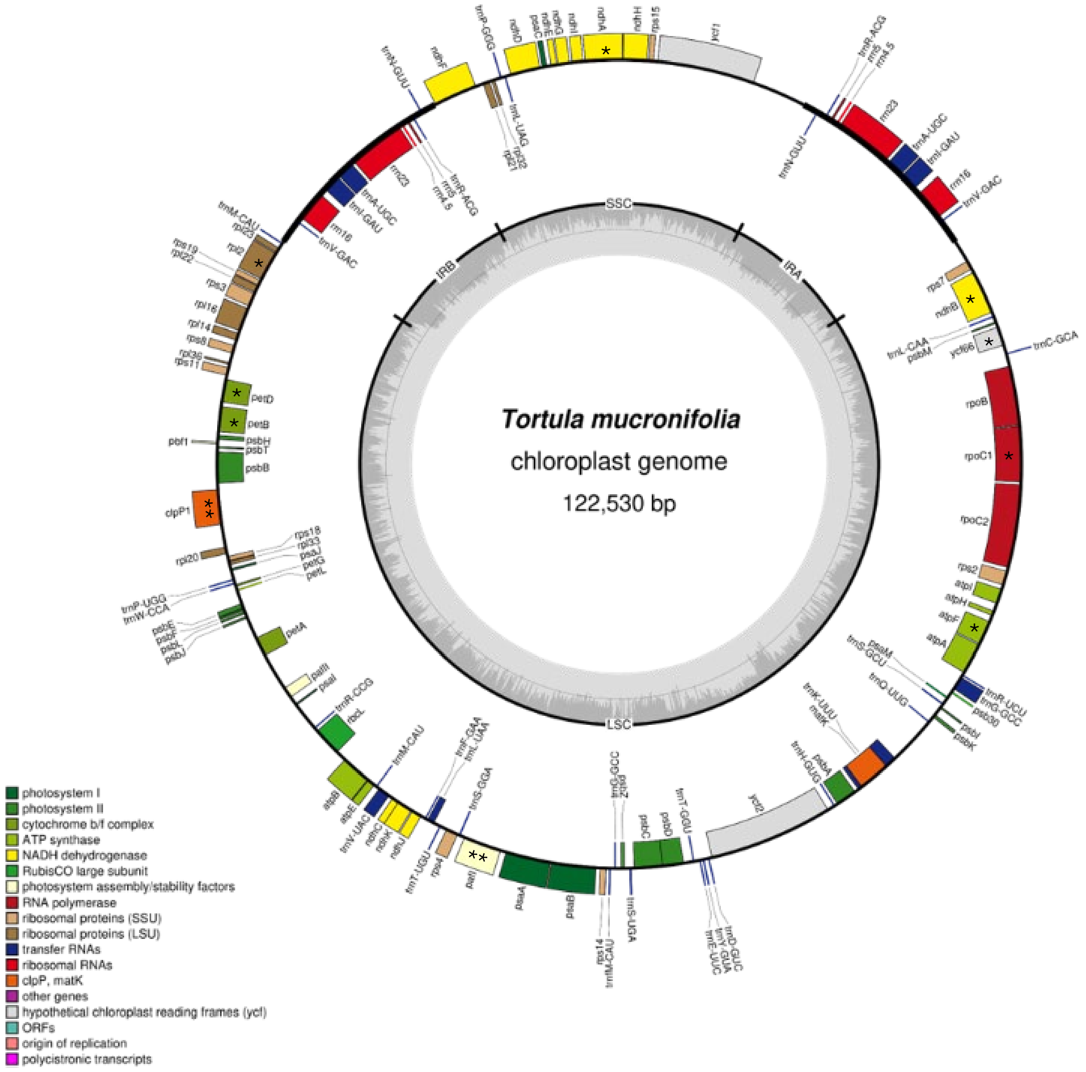

Figure S1.3. Chloroplast genome map of *Tortula mucronifolia* with a total length of 122,530 bp. The inner circle represents the guanine and cytosine (GC) content across different regions of the chloroplast genome and is defined by the major chloroplast regions: large single-copy (LSC), small single-copy (SSC), and inverted repeats (IRA and IRB). The outer circle represents the complete chloroplast sequence, with the genes annotated outside the circle to represent forward strand genes and those annotated inside the circle to represent reversed strand genes. All genes are colored according to their functional groups, as indicated in the right part of the figure (* and ** represent genes with one and two introns, respectively).

d) *Tortula muralis* var. *aestiva*

High-throughput sequencing of *T. muralis*, followed by quality filtering, enabled the successful assembly of the chloroplast genome with a coverage of 127×. The complete chloroplast genome of *T. muralis* is 121,889 bp in length and exhibits a typical quadripartite structure. It consists of LSC region of 83,376 bp, SSC region of 18,653 bp, and a pair of IR regions, each 9,930 bp in length. The overall GC content is 28.40 %, with a nucleotide composition of A (35.9 %), T (35.7 %), C (14.3 %), and G (14.1 %). The chloroplast genome encodes a total of 126 genes, including 81 protein-coding genes, 37 transfer RNAs (tRNAs), and 8 ribosomal RNAs (rRNAs) were identified, consistent with the typical structure of bryophytes plastomes (Figure S1.4).

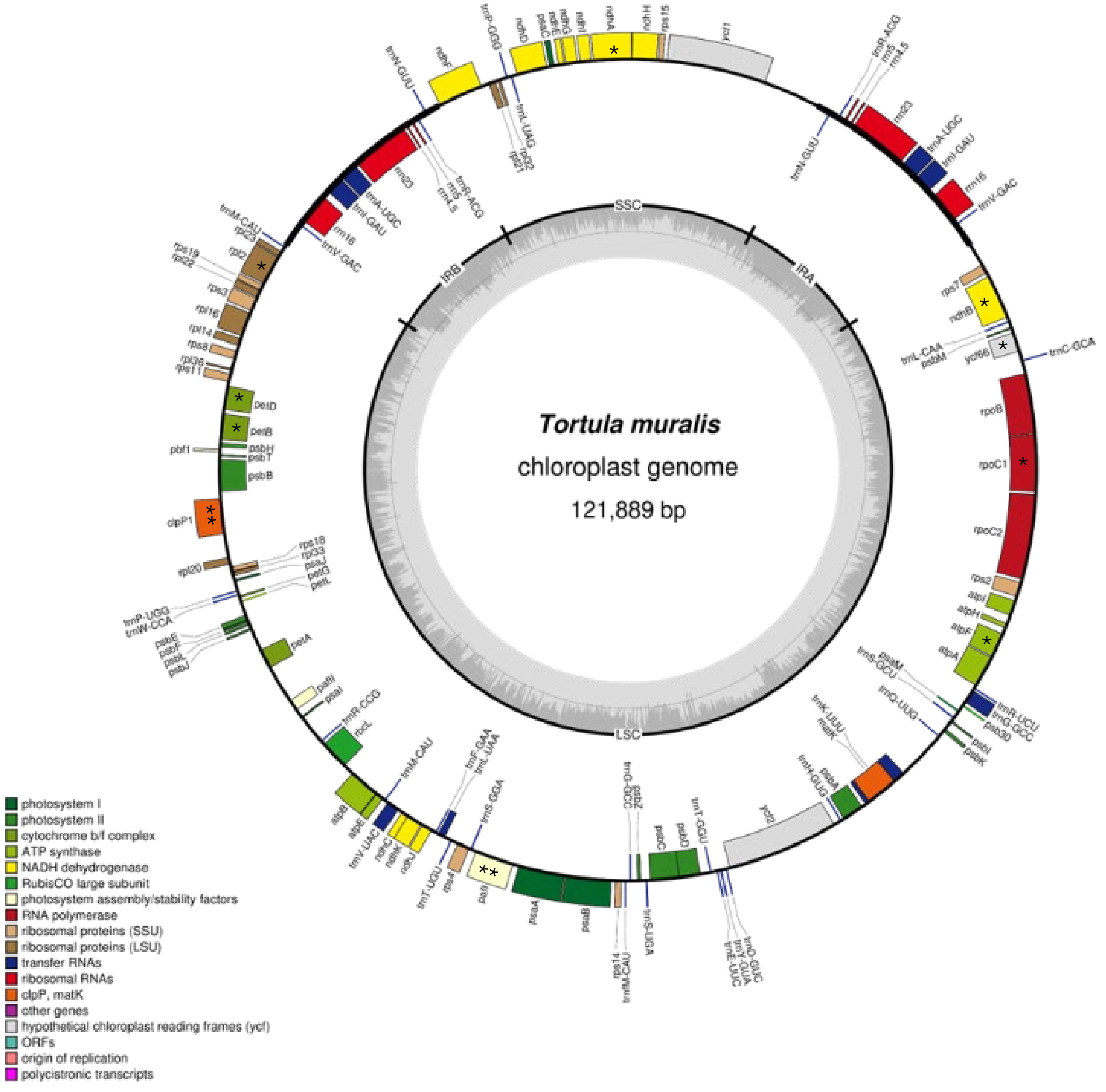

Figure S1.4. Chloroplast genome map of *Tortula muralis* var. *aestiva* with a total length of 121,889 bp. The inner circle represents the guanine and cytosine (GC) content across different regions of the chloroplast genome and is defined by the major chloroplast regions: large single-copy (LSC), small single-copy (SSC), and inverted repeats (IRA and IRB). The outer circle represents the complete chloroplast sequence, with the genes annotated outside the circle to represent forward strand genes and those annotated inside the circle to represent reversed strand genes. All genes are colored according to their functional groups, as indicated in the right part of the figure (* and ** represent genes with one and two introns, respectively).

e) *Tortula subulata*

High-throughput sequencing of *T. subulata*, followed by quality filtering, enabled the successful assembly of the chloroplast genome with a coverage of 428×. The complete chloroplast genome of *T. subulata* is 122,456 bp in length and exhibits a typical quadripartite structure. It consists of a LSC region of 83,914 bp, SSC region of 18,602 bp, and a pair of IR regions, each 9,930 bp in length. The overall GC content is 28.20 %, with a nucleotide composition of A (35.8 %), T (36 %), C (14.2 %), and G (14 %). The chloroplast genome encodes a total of 126 genes, including 81 protein-coding genes, 37 transfer RNAs (tRNAs), and 8 ribosomal RNAs (rRNAs) were identified, consistent with the typical structure of bryophytes plastomes (Figure S1.5).

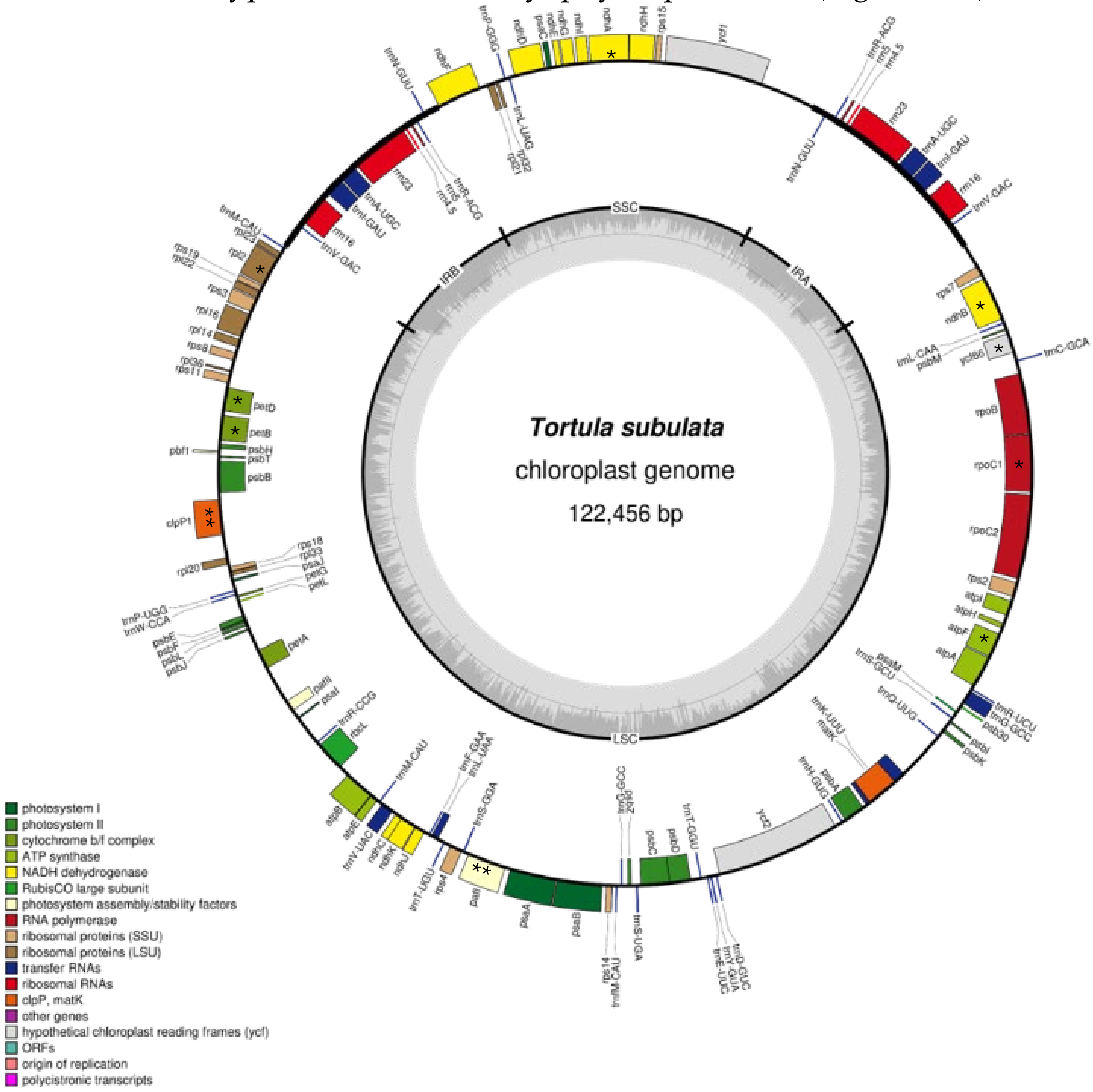

Figure S1.5. Chloroplast genome map of *Tortula subulata* with a total length of 122,456 bp. The inner circle represents the guanine and cytosine (GC) content across different regions of the chloroplast genome and is defined by the major chloroplast regions: large single-copy (LSC), small single-copy (SSC), and inverted repeats (IRA and IRB). The outer circle represents the complete chloroplast sequence, with the genes annotated outside the circle to represent forward strand genes and those annotated inside the circle to represent reversed strand genes. All genes are colored according to their functional groups, as indicated in the right part of the figure (* and ** represent genes with one and two introns, respectively).

2. Section *Cuneifoliae*

a) *Tortula acaulon*

High-throughput sequencing of *T. acaulon*, followed by quality filtering, enabled the successful assembly of the chloroplast genome with a coverage of 147×. The complete chloroplast genome of *T. acaulon* is 122,270 bp in length and exhibits a typical quadripartite structure. It consists of LSC region of 83,864 bp, SSC region of 18,422 bp, and a pair of IR regions, each 9,992 bp in length. The overall GC content is 28.40 %, with a nucleotide composition of A (35.7 %), T (35.9 %), C (14.3 %), and G (14.1 %). The chloroplast genome encodes a total of 126 genes, including 81 protein-coding genes, 37 transfer RNAs (tRNAs), and 8 ribosomal RNAs (rRNAs) were identified, consistent with the typical structure of bryophytes plastomes (Figure S1.6).

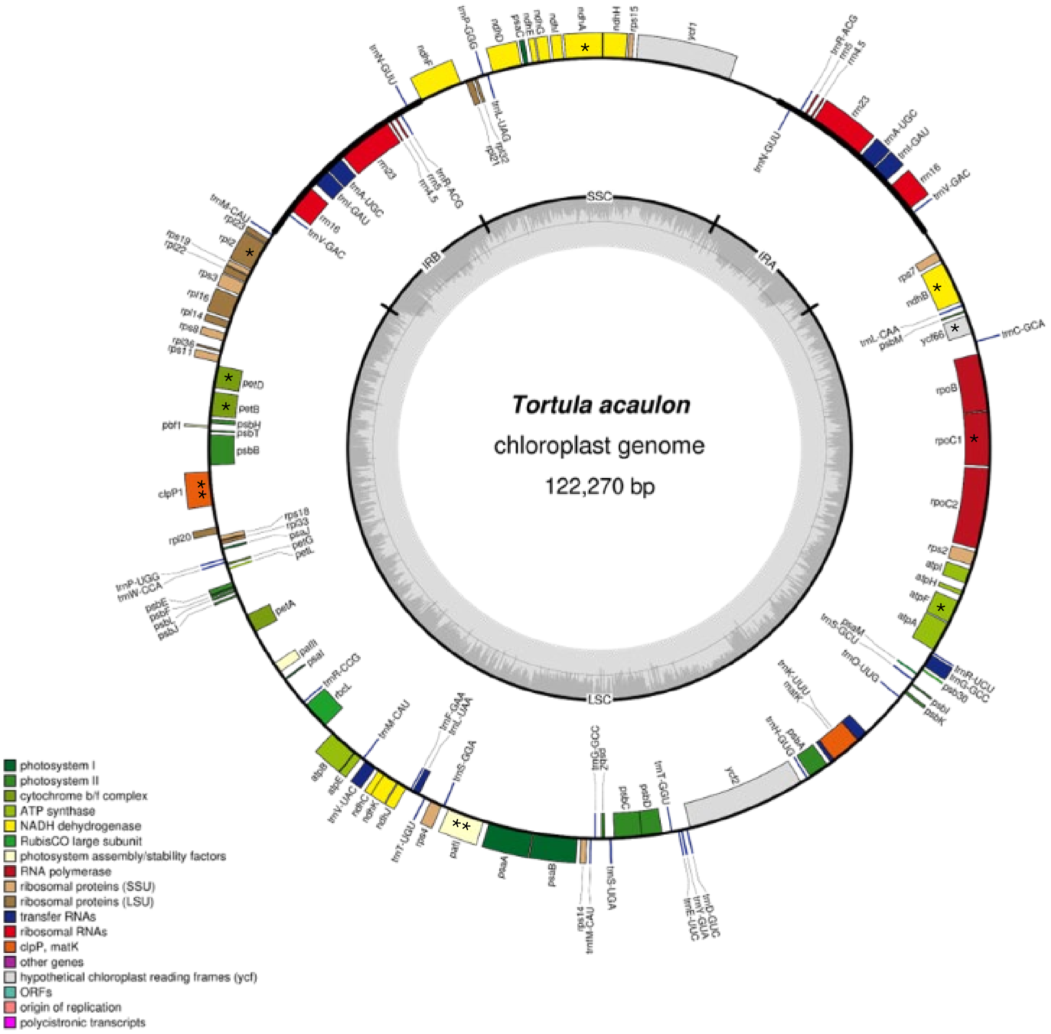

Figure S1.6. Chloroplast genome map of *Tortula acaulon* with a total length of 122,270 bp. The inner circle represents the guanine and cytosine (GC) content across different regions of the chloroplast genome and is defined by the major chloroplast regions: large single-copy (LSC), small single-copy (SSC), and inverted repeats (IRA and IRB). The outer circle represents the complete chloroplast sequence, with the genes annotated outside the circle to represent forward strand genes and those annotated inside the circle to represent reversed strand genes. All genes are colored according to their functional groups, as indicated in the right part of the figure (* and ** represent genes with one and two introns, respectively).

b) *Tortula lindbergii*

High-throughput sequencing of *T. lindbergii*, followed by quality filtering, enabled the successful assembly of the chloroplast genome with a coverage of 182×. The complete chloroplast genome of *T. lindbergii* is 122,515 bp in length and exhibits a typical quadripartite structure. It consists of LSC region of 84,014 bp, SSC region of 18,655 bp, and a pair of IR regions, each 9,923 bp in length. The overall GC content is 28.40 %, with a nucleotide composition of A (35.7 %), T (35.9 %), C (14.3 %), and G (14.1 %).The chloroplast genome encodes a total of 126 genes, including 81 protein-coding genes, 37 transfer RNAs (tRNAs), and 8 ribosomal RNAs (rRNAs) were identified, consistent with the typical structure of bryophytes plastomes (Figure S1.7).

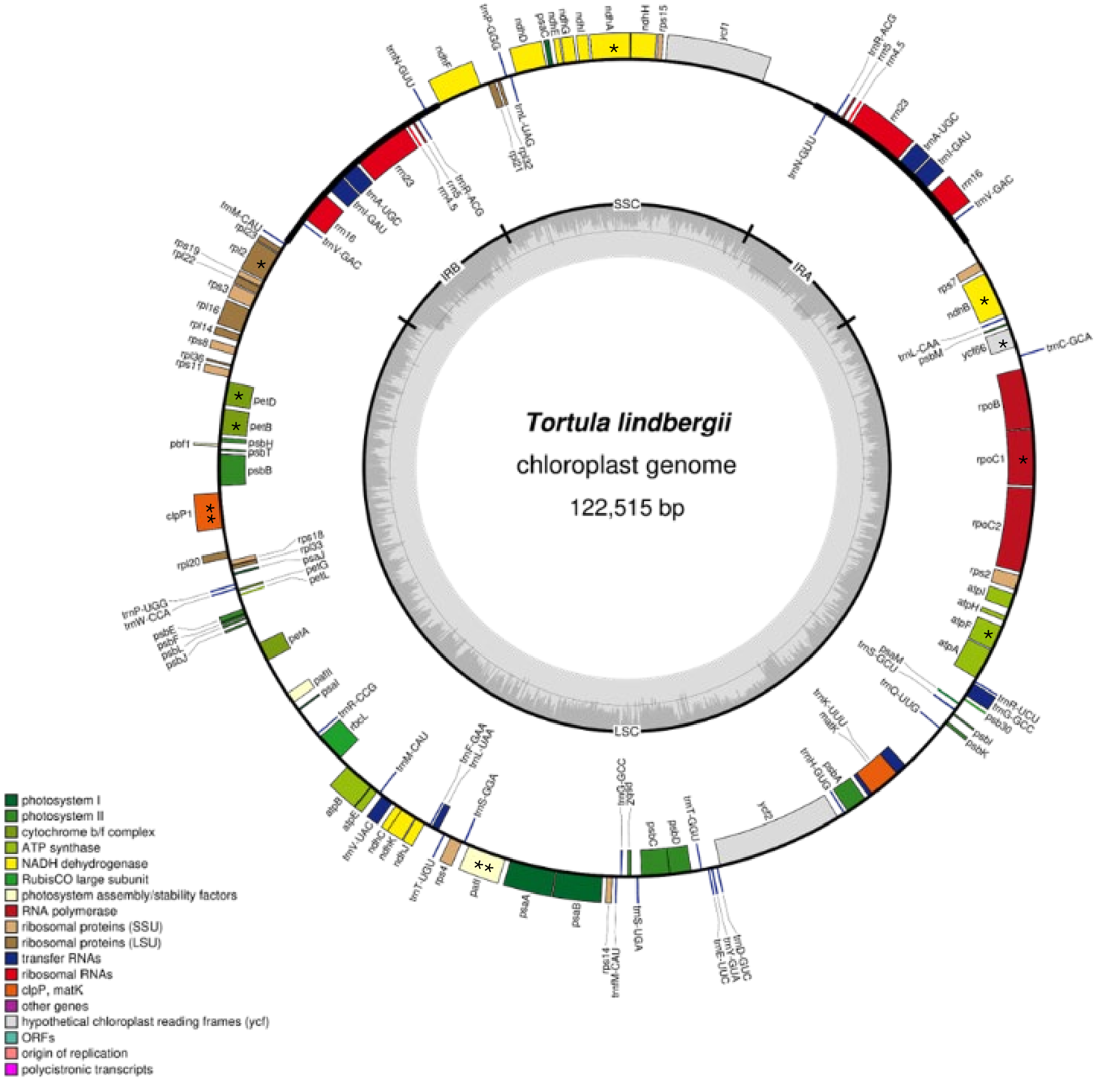

Figure S1.7. Chloroplast genome map of *Tortula lindbergii* with a total length of 122,515 bp. The inner circle represents the guanine and cytosine (GC) content across different regions of the chloroplast genome and is defined by the major chloroplast regions: large single-copy (LSC), small single-copy (SSC), and inverted repeats (IRA and IRB). The outer circle represents the complete chloroplast sequence, with the genes annotated outside the circle to represent forward strand genes and those annotated inside the circle to represent reversed strand genes. All genes are colored according to their functional groups, as indicated in the right part of the figure (* and ** represent genes with one and two introns, respectively).

c) *Tortula pallida*

High-throughput sequencing of *T. pallida*, followed by quality filtering, enabled the successful assembly of the chloroplast genome with a coverage of 1321×. The complete chloroplast genome of *Tortula pallida* is 122,312 bp in length and exhibits a typical quadripartite structure. It consists of LSC region of 83,713 bp, SSC region of 18,595 bp, and a pair of IR regions, each 10,002 bp in length. The overall GC content is 28.50 %, with a nucleotide composition of A (35.8 %), T (35.7 %), C (14.3 %), and G (14.2 %). The chloroplast genome encodes a total of 126 genes, including 81 protein-coding genes, 37 transfer RNAs (tRNAs), and 8 ribosomal RNAs (rRNAs) were identified, consistent with the typical structure of bryophytes plastomes (Figure S1.8).

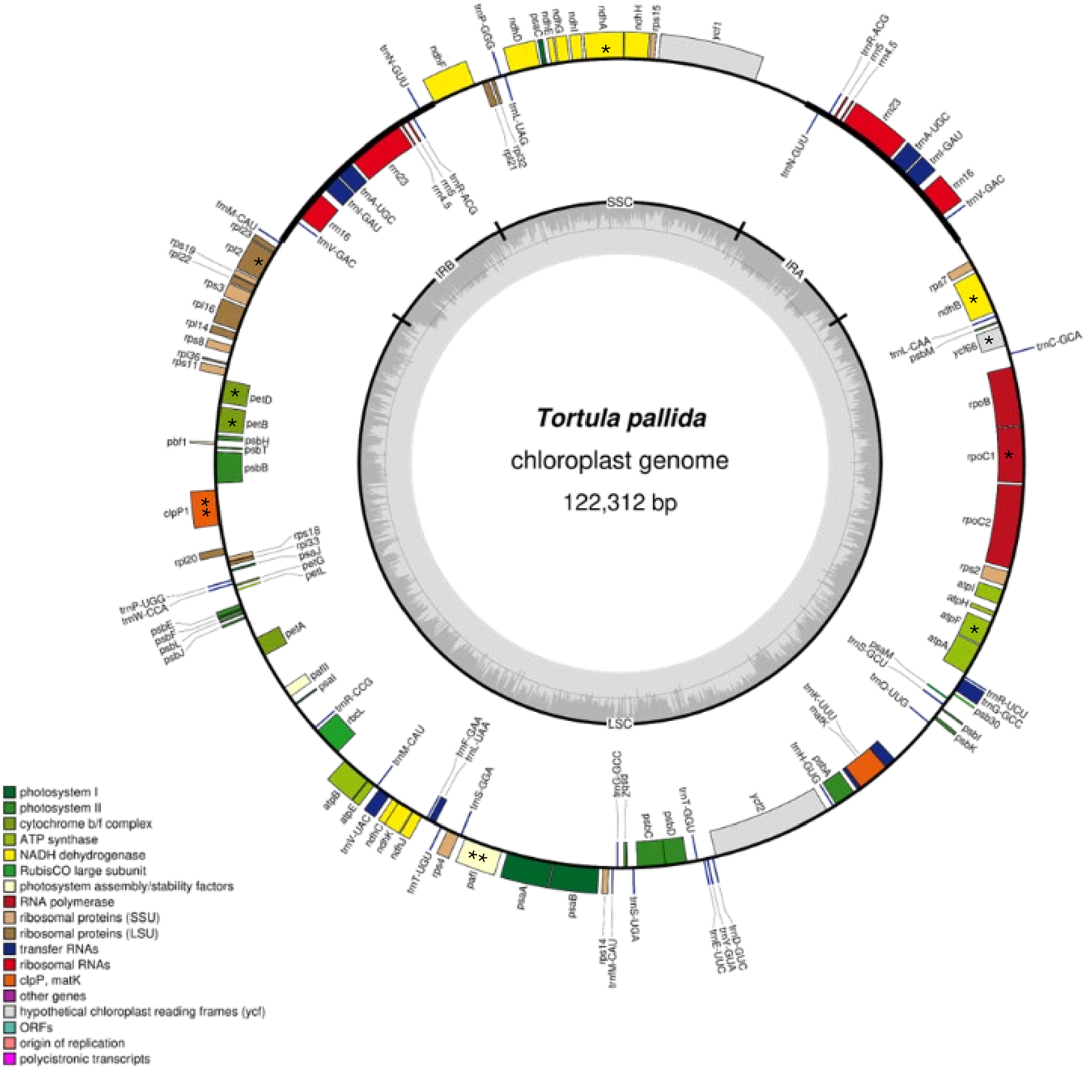

Figure S1.8. Chloroplast genome map of *Tortula pallida* with a total length of 122,312 bp. The inner circle represents the guanine and cytosine (GC) content across different regions of the chloroplast genome and is defined by the major chloroplast regions: large single-copy (LSC), small single-copy (SSC), and inverted repeats (IRA and IRB). The outer circle represents the complete chloroplast sequence, with the genes annotated outside the circle to represent forward strand genes and those annotated inside the circle to represent reversed strand genes. All genes are colored according to their functional groups, as indicated in the right part of the figure (* and ** represent genes with one and two introns, respectively).

d) *Tortula protobryoides*

High-throughput sequencing of *T. protobryoides*, followed by quality filtering, enabled the successful assembly of the chloroplast genome with a coverage of 233×. The complete chloroplast genome of *T. protobryoides* is 122,572 bp in length and exhibits a typical quadripartite structure. It consists of LSC region of 83,987 bp, SSC region of 18,573 bp, and a pair of IR regions, each 10,006 bp in length. The overall GC content is 28.40%, with a nucleotide composition of A (35.7 %), T (35.9 %), C (14.3 %), and G (14.1 %). The chloroplast genome encodes a total of 126 genes, including 81 protein-coding genes, 37 transfer RNAs (tRNAs), and 8 ribosomal RNAs (rRNAs) were identified, consistent with the typical structure of bryophytes plastomes (Figure S1.9).

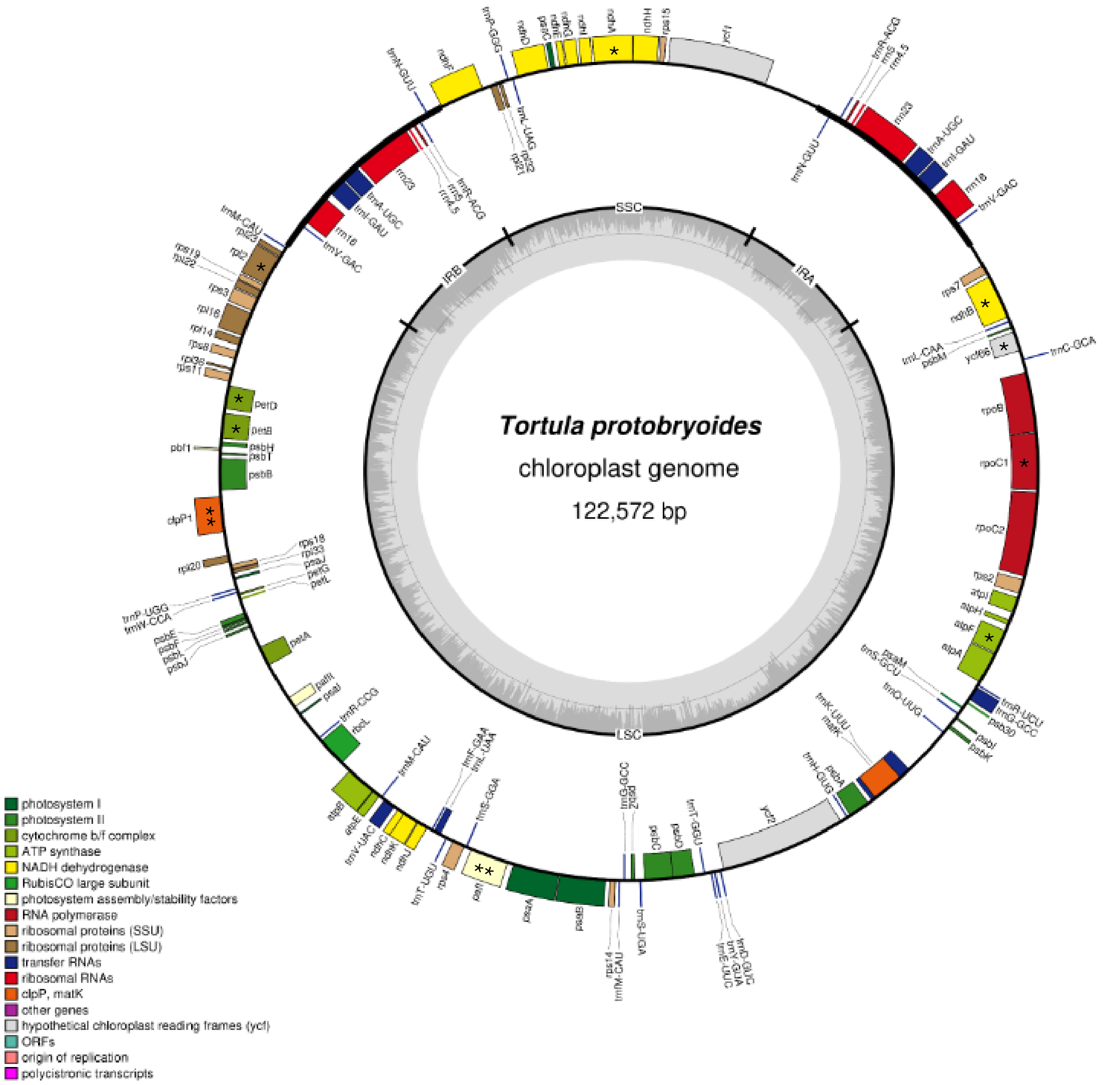

Figure S1.9. Chloroplast genome map of *Tortula protobryoides* with a total length of 122,572 bp. The inner circle represents the guanine and cytosine (GC) content across different regions of the chloroplast genome and is defined by the major chloroplast regions: large single-copy (LSC), small single-copy (SSC), and inverted repeats (IRA and IRB). The outer circle represents the complete chloroplast sequence, with the genes annotated outside the circle to represent forward strand genes and those annotated inside the circle to represent reversed strand genes. All genes are colored according to their functional groups, as indicated in the right part of the figure (* and ** represent genes with one and two introns, respectively).

3. Outgroup species: *Syntrichia princeps*

High-throughput sequencing of S. *princeps*, followed by quality filtering, enabled the successful assembly of the chloroplast genome with a coverage of 490×. The complete chloroplast genome of *S. princeps* is 122,562 bp in length and exhibits a typical quadripartite structure. It consists of LSC region of 84,045 bp, SSC region of 18,547 bp, and a pair of IR regions, each 9,985 bp in length. The overall GC content is 28.30 %, with a nucleotide composition of A (35.8 %), T (35.9 %), C (14.2 %), and G (14.1 %). The chloroplast genome encodes a total of 126 genes, including 81 protein-coding genes, 37 transfer RNAs (tRNAs), and 8 ribosomal RNAs (rRNAs) were identified, consistent with the typical structure of bryophytes plastomes (Figure S1.10).

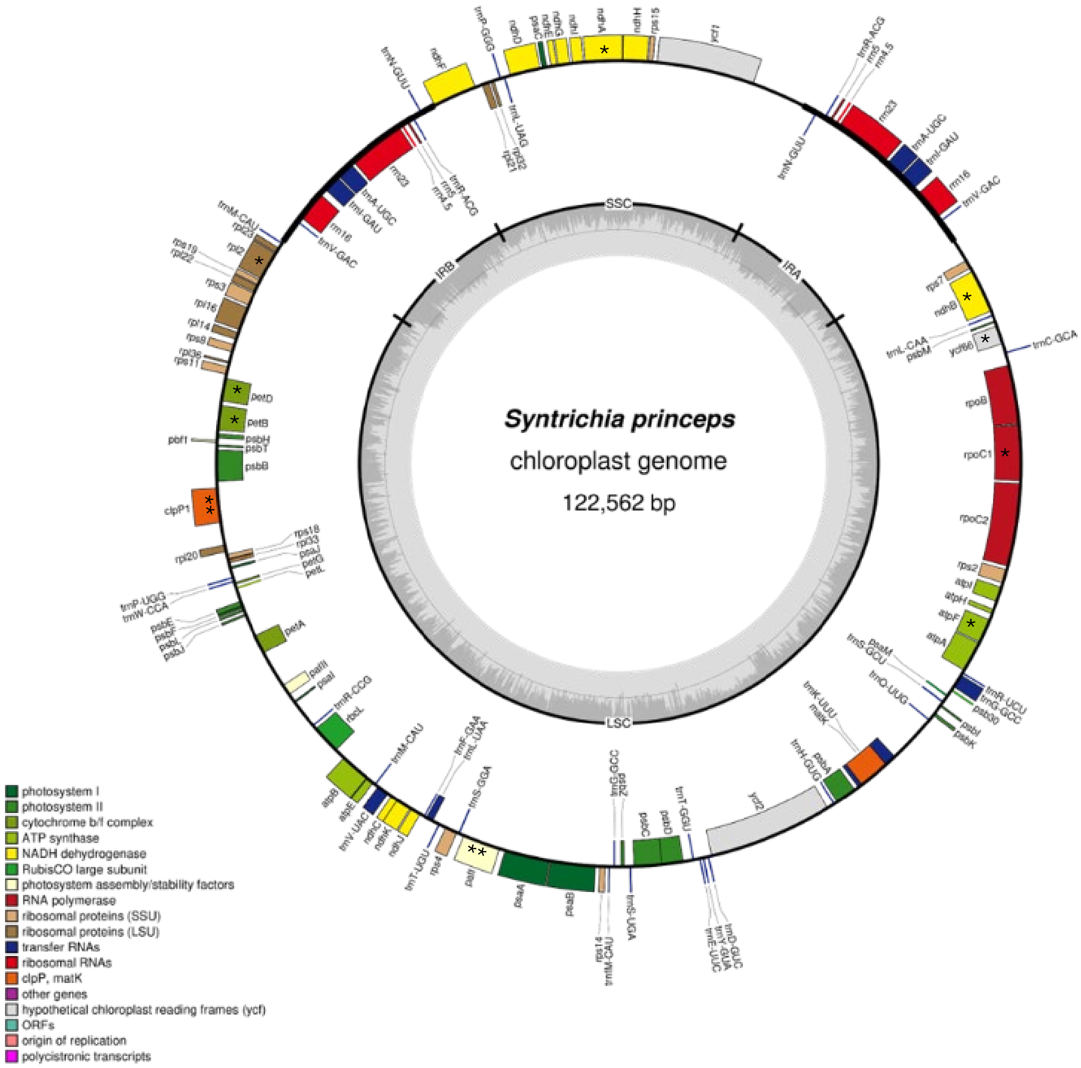

Figure S1.10. Chloroplast genome map of *Syntrichia princeps* with a total length of 122,562 bp. The inner circle represents the guanine and cytosine (GC) content across different regions of the chloroplast genome and is defined by the major chloroplast regions: large single-copy (LSC), small single-copy (SSC), and inverted repeats (IRA and IRB). The outer circle represents the complete chloroplast sequence, with the genes annotated outside the circle to represent forward strand genes and those annotated inside the circle to represent reversed strand genes. All genes are colored according to their functional groups, as indicated in the right part of the figure (* and ** represent genes with one and two introns, respectively).
