# Supplementary material for: Exploring Plastome Diversity and Molecular Evolution Within Genus Tortula (Family Pottiaceae, Bryophyta)
Source: Plants (Basel). 2025 Sep 8;14(17):2808. doi: 10.3390/plants14172808 (PMC12430682; doi:10.3390/plants14172808)
Supplement: Supplementary file 1 [file plants-14-02808-s001.zip › Figures S2.pdf]

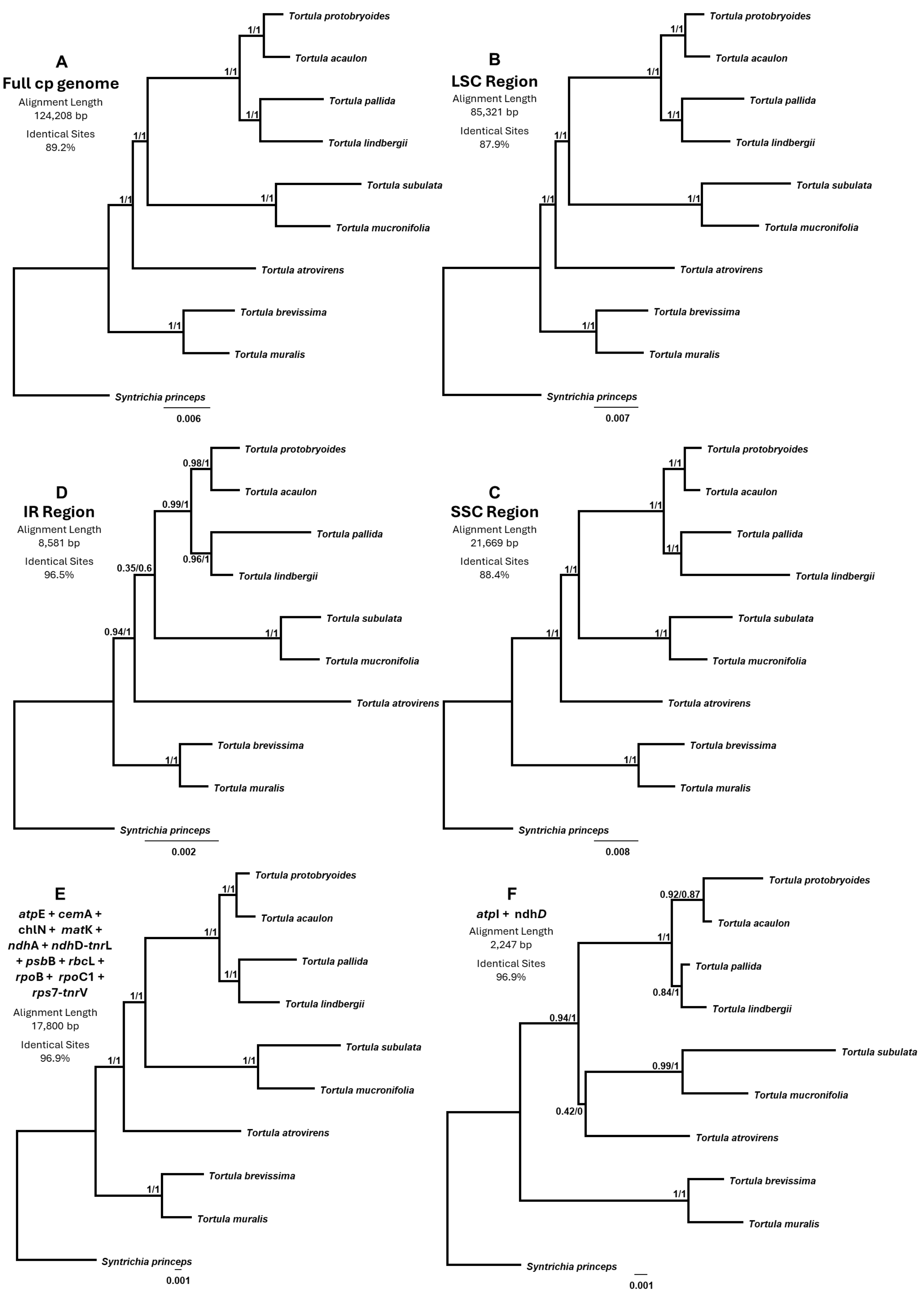
A
Full cp genome
Alignment Length
124,208 bp
Identical Sites
89.2%
Tortula protobryoides
Tortula acaulon
Tortula pallida
Tortula lindbergii
Tortula subulata
Tortula mucronifolia
Tortula atrovirens
Tortula brevissima
Tortula muralis
Syntrichia princeps
1/1
0.006
B
LSC Region
Alignment Length
85,321 bp
Identical Sites
87.9%
0.007
D
IR Region
Alignment Length
8,581 bp
Identical Sites
96.5%
0.98/1
0.99/1
0.96/1
0.35/0.6
0.94/1
0.002
C
SSC Region
Alignment Length
21,669 bp
Identical Sites
88.4%
0.008
E
atpE + cemA + chlN + matK + ndhA + ndhD-tnrL + psbB + rbcL + rpoB + rpoC1 + rps7-tnrV
Alignment Length
17,800 bp
Identical Sites
96.9%
0.001
F
atpI + ndhD
Alignment Length
2,247 bp
Identical Sites
96.9%
0.92/0.87
0.84/1
0.94/1
0.99/1
0.42/0
0.001

Figure S2.1. Phylogenetic trees reconstructed from full plastome and selected loci of *Tortula* species using Maximum Likelihood (ML) and Bayesian Inference (BI) methods. Topologies are based on: (A) complete plastome sequences, (B) Large single-copy (LSC) region, (C) Small single-copy (SSC) region, (D) Inverted repeat (IR) regions, (E) concatenated hypervariable regions supporting full plastome signal, and (F) *atp*I and *ndh*D loci that deviate in the phylogenetic placement of *Tortula atrovirens*. Support values on branches indicate ML bootstrap values followed by BI posterior probabilities (ML/BI) (shown in decimal 0-1). Scale indicates genetic distance.

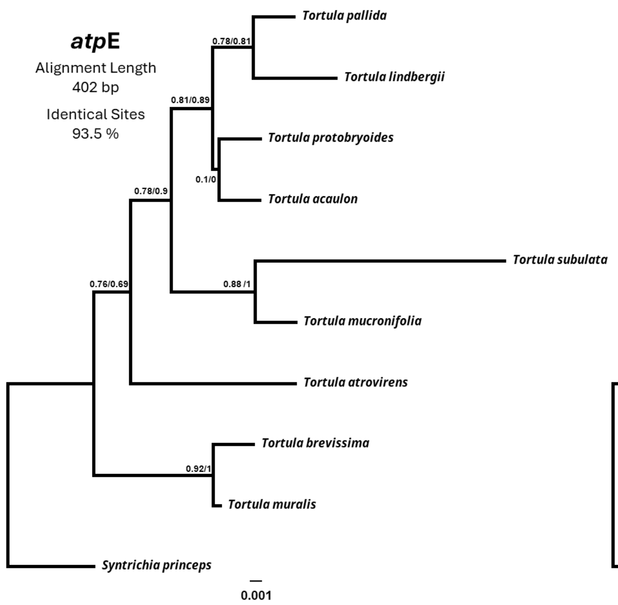
atpE
Alignment Length
402 bp
Identical Sites
93.5 %
0.78/0.81
0.81/0.89
0.1/0
0.78/0.9
0.88 /1
0.76/0.69
0.92/1
Tortula pallida
Tortula lindbergii
Tortula protobryoides
Tortula acaulon
Tortula subulata
Tortula mucronifolia
Tortula atrovirens
Tortula brevissima
Tortula muralis
Syntrichia princeps
0.001

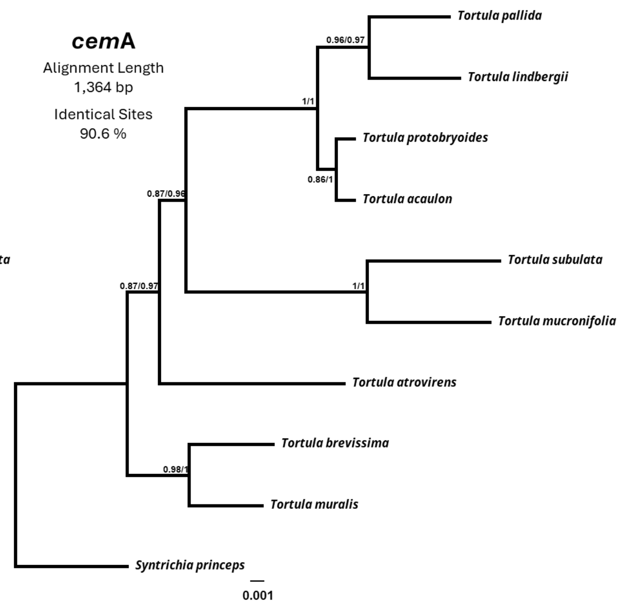
cemA
Alignment Length
1,364 bp
Identical Sites
90.6 %
0.96/0.97
1/1
0.86/1
0.87/0.98
0.87/0.97
1/1
0.98/1
Tortula pallida
Tortula lindbergii
Tortula protobryoides
Tortula acaulon
Tortula subulata
Tortula mucronifolia
Tortula atrovirens
Tortula brevissima
Tortula muralis
Syntrichia princeps
0.001

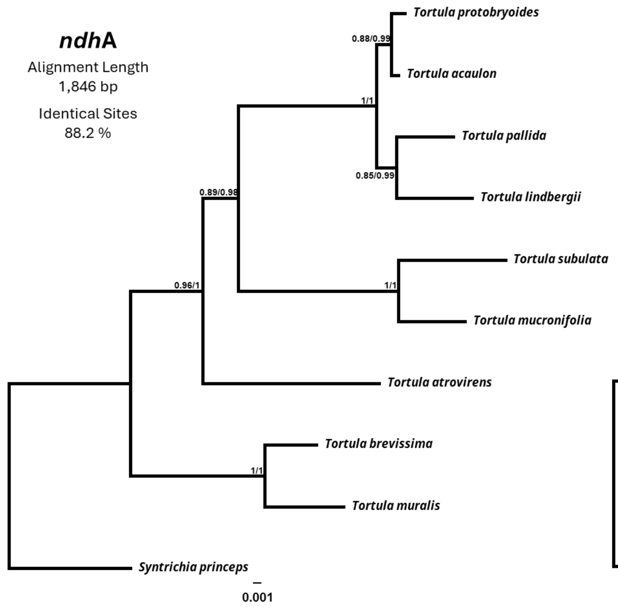
ndhA
Alignment Length
1,846 bp
Identical Sites
88.2 %
0.88/0.99
1/1
0.85/0.99
0.89/0.98
0.96/1
1/1
1/1
Tortula protobryoides
Tortula acaulon
Tortula pallida
Tortula lindbergii
Tortula subulata
Tortula mucronifolia
Tortula atrovirens
Tortula brevissima
Tortula muralis
Syntrichia princeps
0.001

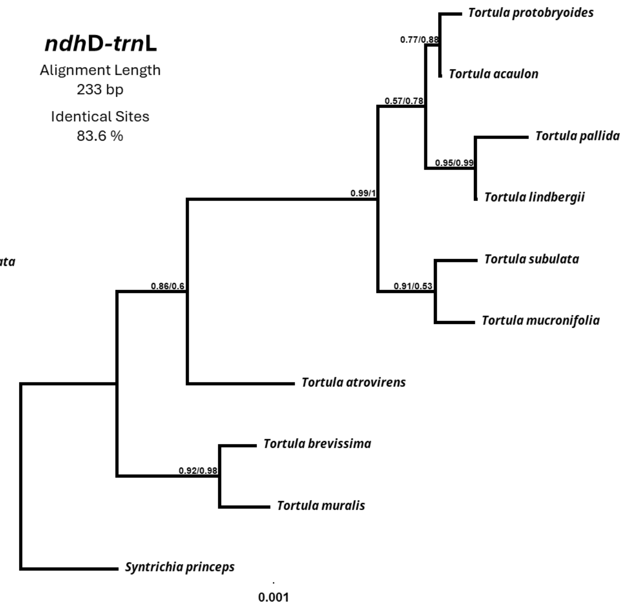
ndhD-trnL
Alignment Length
233 bp
Identical Sites
83.6 %
0.77/0.88
0.57/0.78
0.95/0.99
0.99/1
0.86/0.6
0.91/0.53
0.92/0.98
Tortula protobryoides
Tortula acaulon
Tortula pallida
Tortula lindbergii
Tortula subulata
Tortula mucronifolia
Tortula atrovirens
Tortula brevissima
Tortula muralis
Syntrichia princeps
0.001

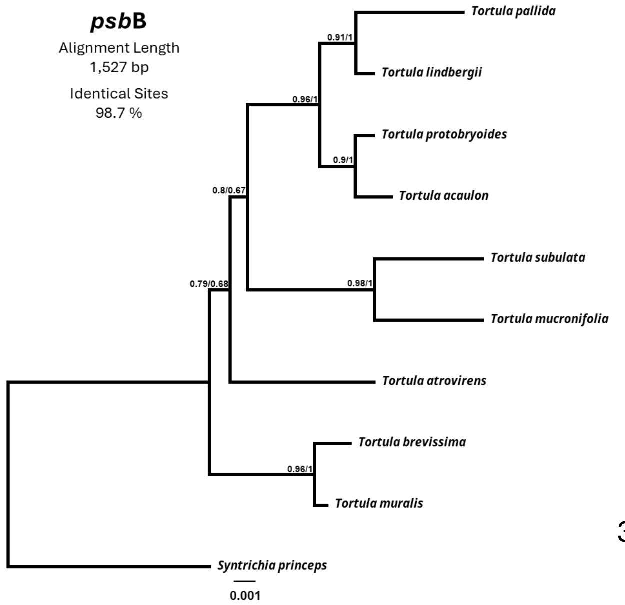
psbB
Alignment Length
1,527 bp
Identical Sites
98.7 %
0.91/1
0.96/1
0.9/1
0.8/0.67
0.98/1
0.79/0.68
0.96/1
Tortula pallida
Tortula lindbergii
Tortula protobryoides
Tortula acaulon
Tortula subulata
Tortula mucronifolia
Tortula atrovirens
Tortula brevissima
Tortula muralis
Syntrichia princeps
0.001

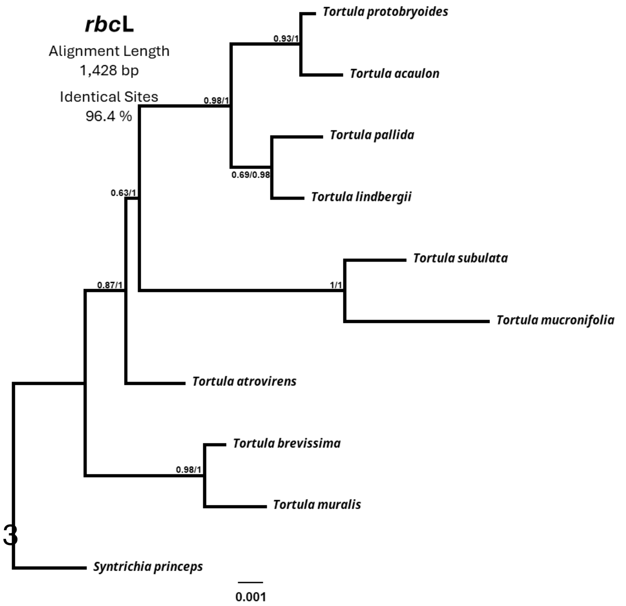
rbcL
Alignment Length
1,428 bp
Identical Sites
96.4 %
0.93/1
0.98/1
0.69/0.98
0.63/1
1/1
0.87/1
0.98/1
Tortula protobryoides
Tortula acaulon
Tortula pallida
Tortula lindbergii
Tortula subulata
Tortula mucronifolia
Tortula atrovirens
Tortula brevissima
Tortula muralis
Syntrichia princeps
0.001

Figure S2.2. Individual loci phylogenetic trees reconstructed based on selected loci (group S2.1E) of *Tortula* species using Maximum Likelihood (ML) and Bayesian Inference (BI) methods. Support values on branches indicate ML bootstrap values followed by BI posterior probabilities (ML/BI) (shown in decimal 0-1). Scale indicates genetic distance.

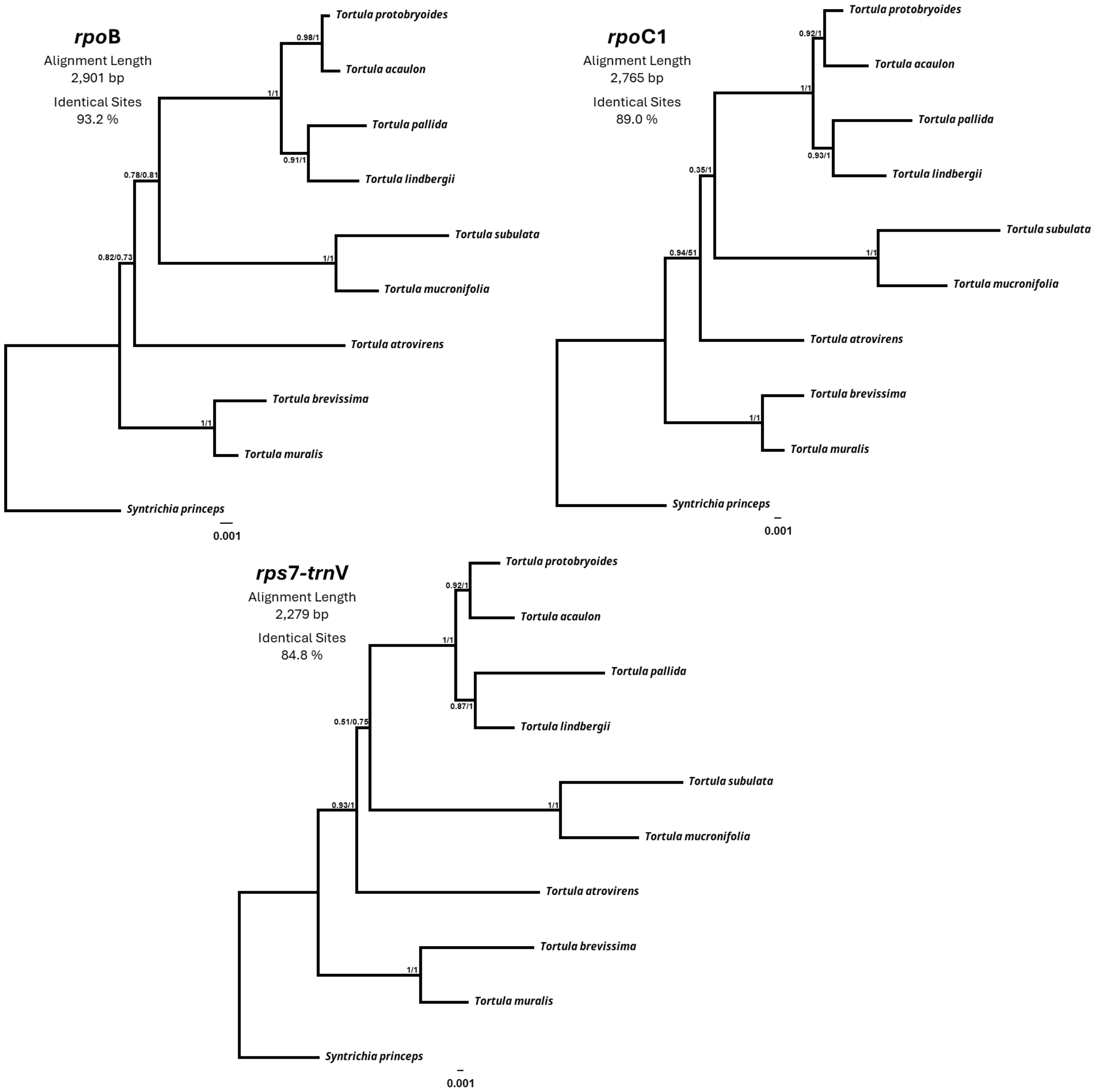

Figure S2.2. Continue.

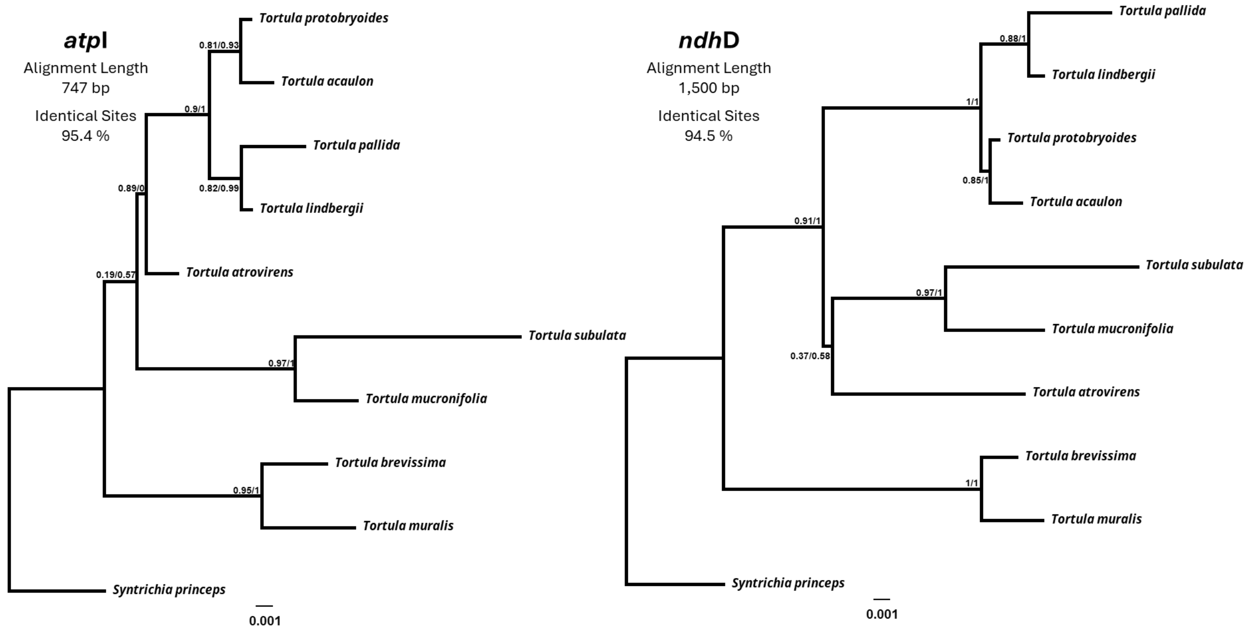

Figure S2.3. Individual loci phylogenetic trees reconstructed based on selected loci (group S2.1F) of *Tortula* species using Maximum Likelihood (ML) and Bayesian Inference (BI) methods. Support values on branches indicate ML bootstrap values followed by BI posterior probabilities (ML/BI) (shown in decimal 0-1). Scale indicates genetic distance.
