# Supplementary material for: Exploring Plastome Diversity and Molecular Evolution Within Genus Tortula (Family Pottiaceae, Bryophyta)
Source: Plants (Basel). 2025 Sep 8;14(17):2808. doi: 10.3390/plants14172808 (PMC12430682; doi:10.3390/plants14172808)
Supplement: Supplementary file 1 [file plants-14-02808-s001.zip › Tabla S4.pdf]

Table S4. Collected plant material: species, location, and voucher information, including geographical coordinates, habitat, altitude, collection date, collector's name, MUB herbarium code, and sample ID number.

Species name	Location name	Geographical coordinates	Habitat	Altitude (m a.s.l.)	Collection date	Collectors name	MUB herbarium code	Sample ID number
***Syntrichia princeps* (De Not.) Mitt.**	SPAIN: Teruel province, Puerto El Portillo, carretera TE-V-9032	40.368758 N, 1.737420 W	Small siliceous slope on the hillside of a *Pinus sylvestris* forest	1760	27/06/2023	*R.M. Ros, O. Werner s.n.*	63606	409
Tortula acaulon* (With.) R.H. Zander var. *acaulon	SPAIN: Jaén province, Sierra Sur de Jaén, Monte de las Ánimas, alrededores de Casas Rurales Monte de las Ánimas	37.59221 N, 3.77747 W	Soil on the shoulder of a forest road, protected by large bushes	1277	04/05/2021	*R.M. Ros, O. Werner s.n.*	63597	473
***Tortula atrovirens* (Sm.) Lindb.**	SPAIN: Albacete province, Sierra del Relumbrar, carretera AB-5032 km 12, en su cruce con el río Guadalmena	38.554972 N, 2.731029 W	Soil layer over siliceous rock in a *Quercus rotundifolia* forest	703	11/04/2023	*R.M. Ros, O. Werner s.n.*	63598	234
***Tortula brevissima* Schiffn.**	SPAIN: Murcia province, carretera RM-503 km 28, entre Zarzadilla de Totana y Bullas	37.942385 N, 1.667403W	Sandy soil layer at the bottom of a dry water channel at the roadside	853	30/03/2023	*R.M. Ros, O. Werner s.n.*	63599	70
***Tortula lindbergii* Broth.**	SPAIN: Albacete province, carretera AB-603 km 3, de Alcaraz a Canaleja	38.695756 N, 2.517862 W	Basic, exposed soil in open shrubland dominated by *Retama sphaerocarpa*	928	12/04/2023	*R.M. Ros, O. Werner s.n.*	63600	248
***Tortula mucronifolia* Schwägr.**	SPAIN: Murcia province, subida al Pico del Obispo	38.071260 N, 2.268584 W	Basic soil accumulated under a *Pinus nigra* root	1814	01/08/2023	*R.M. Ros, O. Werner s.n.*	63601	444
***Tortula muralis* Hedw. var. *aestiva* Brid. ex Hedw.**	SPAIN: Murcia province, San Pedro del Pinatar	37.82773 N, 0.80295 W	Soil accumulated on a shaded artificial wall	20	26/03/2023	*F. Alcaraz s.n.*	63602	2
***Tortula pallida* Lindb.**	SPAIN: Albacete province, Carretera AB-4000, a 1 kn de Agramón desde Estación de Agramón	38.412316 N, 1.628548 W	Marly soil accumulated over the concrete of a bridge at the edge of a salt marsh	384	10/04/2023	*R.M. Ros, O. Werner s.n.*	63603	265
***Tortula protobryoides* R.H. Zander**	SPAIN: Murcia province, Moratalla, Casicas del Portal, 500 m south of the hamlet	38.22570 N, 2.05109 W	Basic soil among herbaceous plants under *Juniperus phoenicea* shrub	1263	06/04/2021	*R.M. Ros, O. Werner s.n.*	63604	40
***Tortula subulata* Hedw.**	SPAIN: Guadalajara province, carretera GU-982, cruce con río Tajo, Las Tres Cruces	40.397996 N, 1.805469 W	Sandy siliceous soil accumulated at the base of a *Pinus sylvestris* tree	1507	27/06/2023	*R.M. Ros, O. Werner s.n.*	63605	395
